# Supplementary figures and images for: Stratified analysis of the association between periodontitis and female breast cancer based on age, comorbidities and level of urbanization: A population-based nested case-control study
Source: PLoS One. 2022 Jul 26;17(7):e0271948. doi: 10.1371/journal.pone.0271948 (PMC9321417; doi:10.1371/journal.pone.0271948)

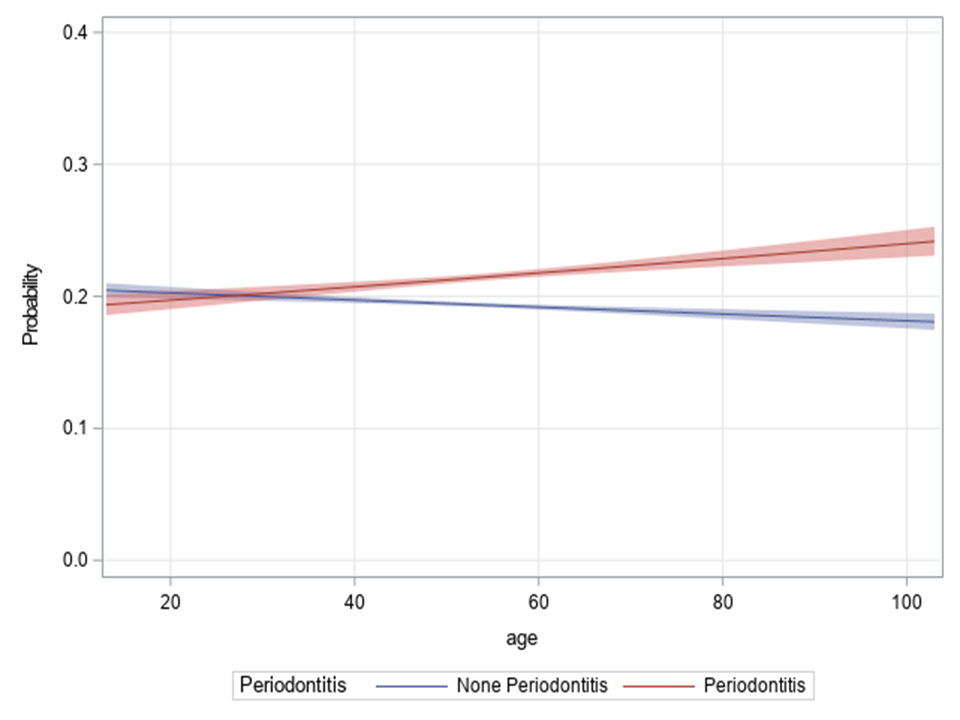

Supplement: S1 Fig — (TIF) [file pone.0271948.s001.tif]

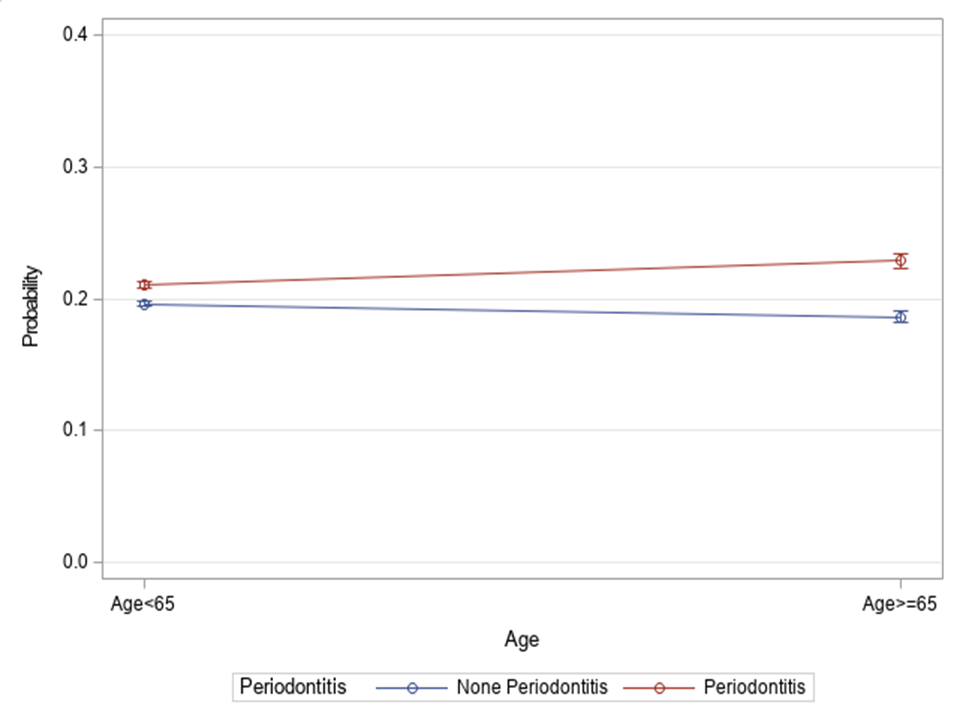

Supplement: S2 Fig — (TIF) [file pone.0271948.s002.tif]

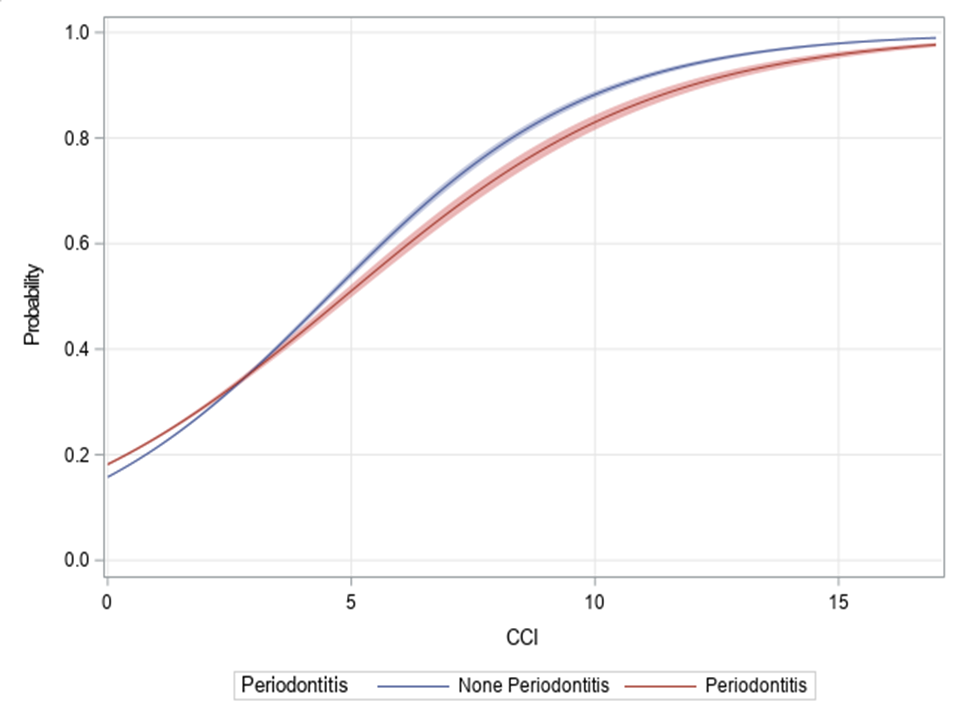

Supplement: S3 Fig — (TIF) [file pone.0271948.s003.tif]

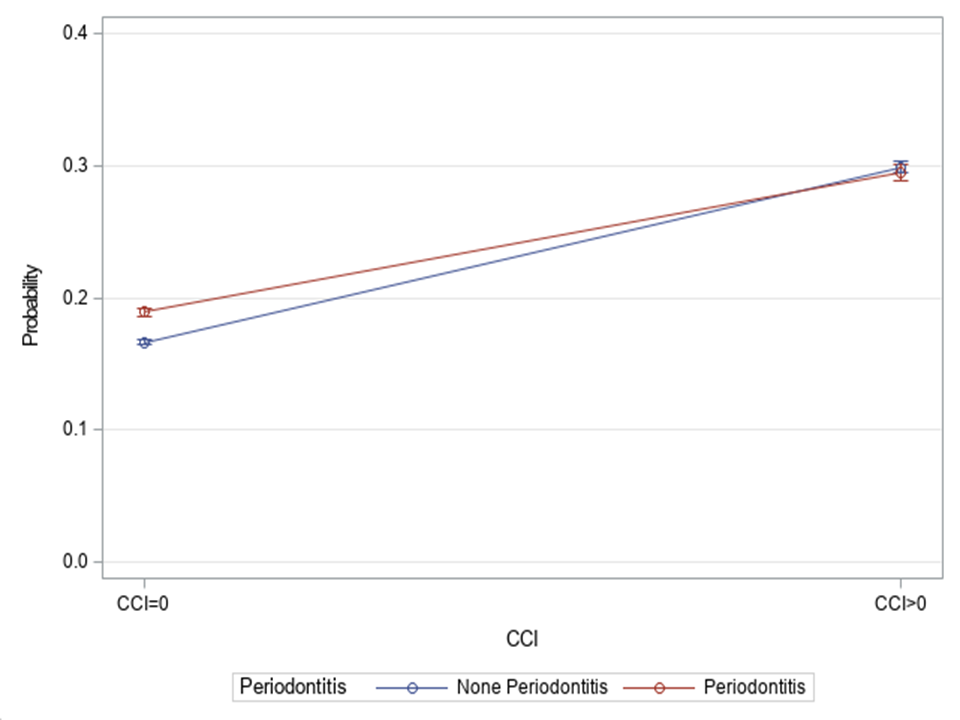

Supplement: S4 Fig — (TIF) [file pone.0271948.s004.tif]
