# Supplementary material for: Stratified analysis of the association between periodontitis and female breast cancer based on age, comorbidities and level of urbanization: A population-based nested case-control study
Source: PLoS One. 2022 Jul 26;17(7):e0271948. doi: 10.1371/journal.pone.0271948 (PMC9321417; doi:10.1371/journal.pone.0271948)
Supplement: Attachment — Submitted filename: response to reviewers 20220609.docx [file pone.0271948.s005.docx]

Reviewer #1: Introduction
1.Page 4. Did the prevalence of periodontitis increase because of changes in definitions? This has often been the case in other countries.
Response: Thanks for your comment! Yes, the prevalence of periodontitis varied with changes in definitions based on ICD-9-CM codes (Table 1). Therefore, we conducted a sensitivity analysis for the association between periodontitis and breast cancer using various definitions of periodontitis and found consistent results with ORs from 1.10 to 1.12. Also, the non-differential misclassification bias related to periodontitis diagnosis always drives the bias towards the null. Therefore, the magnitude of the association between periodontitis and breast cancer could only be underestimated. We added a description regarding the influence of periodontitis definition on its association with breast cancer in the last paragraph of the Discussion section: ‘Third, although the Bureau of NHI audit had improved coding accuracy, the accuracy of diagnoses based on claims data is still an issue of concern. However, the non-differential misclassification bias related to periodontitis diagnosis always drives the bias towards the null. Therefore, the magnitude of the association between periodontitis and breast cancer could only be underestimated.’

2.Page 4. There are many other cohort studies on periodontitis and breast cancer that are not referenced (e.g. Farhat et al CEBP 2021, Jia et al CEBP 2020, and earlier meta-analysis of 11 studies: Shao et al 2018). Also, the Sfreddo et al study referenced is not a cohort study. Please edit the last paragraph of the introduction to reflect this. The discussion (first paragraph) also needs to be revised since the association between periodontal disease and breast cancer risk has previously been reported in Taiwan using the same dataset (PMID: 26280747); please explain the differences/overlap between the two studies in the discussion section.
Response: Shao et al [21] demonstrated a meta-analysis study and found periodontal disease may be a risk of female breast cancer. (page 4)

A meta-analysis study [21] found a potential association between periodontitis and breast cancer. (page 11)

In contrast, we analyzed the association between periodontitis and breast cancer in females, and we also found the important modification effects by age and comorbidities. (page 11)

3.Methods
This is not a traditional case-control study; based on the selection of all breast cancer cases during a 10-year period and selection of controls from a large cohort database for comparison, this would be better described as a retrospective population-based cohort study.
Response: Because we retrospectively selected the population of interested based on the outcome (i.e., incident female breast cancer patients as cases and female individuals without breast cancer as controls) and identified the history of periodontitis exposure before the index date, this study was a retrospective population-based case-control study. (page 5)

4.It appears that all incident breast cancer cases identified through the NHIRI between 2003 and 2013 were included in this analysis. It is unclear if the breast cancer cases that are not in the NHRI database would have detailed data on claims for periodontal disease or other co-morbidities and if these were also included. A figure would help between understand the selection process including exclusions that were made and available data available for each step.
Response: Taiwan's National Health Insurance (NHI) began in March 1, 1995 and covered 99.9% of Taiwan's residents by 2014. If the female breast cancer cases were not included in the NHRI database, data on claims for periodontal disease or other comorbidities should be unavailable. We added a figure to show the process of study subjects inclusion.

5.Statistical analysis – please provide details on how the tests for interactions were conducted.
Response: We had provided details on how to test the interaction effects in the statistical method subsection in the Methods section: ‘The significance of modification effect by each covariate on periodontitis exposure-associated female breast cancer risk was examined by calculating the *P*-value of the coefficient associate with the product of each indicator of the covariate and the indicator of periodontitis using the Wald test.’ (page 9)

6.Would it be possible to differentiate different types of breast cancers? E.g. invasive vs non-invasive?

Response: Female breast cancer patients were defined as those having a Catastrophic Illness Certificate (CIC) for breast cancer [International Classification of Diseases, Ninth Revision, Clinical Modification (ICD-9-CM) code 174]. This criteria only included the invasive breast cancer patients.

7.Also, given the strong association between urbanization and breast cancer, it would be useful to examine whether the periodontal disease and breast cancer is modified by this variable (this could be added to Table 5).
Response: We have examined the modification effect of urbanization on the association between periodontitis and female breast cancer and added the results to Table 5.

8.Discussion
The discussion could be improved. Prior results from existing studies on this topic are not provided (e.g., range of RRs to compare magnitude of association, quality of periodontal disease measurement). It would also be helpful to point out some limitations in prior studies, and what this study adds to the literature (given that there are over 10 papers on this topic). A better synthesis of the literature is needed.
Response: Soder *et al*. [20] selected 3,273 patients aged between 30-40 years and found that chronic periodontitis indicated by missing molars seemed to be associated with breast cancer (odds ratio 2.36). Of note, a meta-analysis study [21] found a periodontal disease significantly increased breast cancer risk (RR = 1.22, 95% CI: 1.06-1.40). However, the risk of breast cancer development was not significantly increased among patients with periodontal disease and a history of periodontal therapy. Therefore, the author concluded a potential association between periodontitis and breast cancer. Freudenheim *et al*. [24] analyzed a cohort of 73,737 postmenopausal women and also revealed that periodontitis was associated with the risk of postmenopausal breast cancer (HR 1.14, 95% CI: 1.03-1.26). On the contrary, Farhat *et al*. [25] revealed no association between periodontal disease and overall breast cancer risk (HR 1.02, 95% CI: 0.94-1.10). However, in this study, no confounder was analyzed. In contrast, we analyzed the association between periodontitis and breast cancer adjusting for potential confounds, and we also found the important modification effects by age and comorbidities. (page 11)

9.Typos (tables and “CRT” in discussion) and English grammar need to be corrected throughout.

Response: We corrected it. (page 13)

Reviewer #2: This study explored the effect of periodontitis on female breast cancer using a population based case control study. The paper is reasonably well written though I have some comments which the authors might consider to improve their study.

10.Introduction
Page 4 final paragraph – "small sample size" is somewhat relative – I would state the sample sizes of these studies
Response: Soder et al. [20] analyzed 3,273 patients and revealed that severe periodontal disease increased the risk of breast cancer. (page 4)

11.Page 5: "...while Sfreddo et al. [21] found there was a significant association between periodontitis and breast cancer" - Presumably this is a positive association? Please clarify in the text
Response: We clarified the results of the meta-analysis study by Sfreddo et al. [21]: ‘ Of note, a meta-analysis study [21] found a periodontal disease significantly increased breast cancer risk. However, the risk of breast cancer development was not significantly increased among patients with periodontal disease and a history of periodontal therapy. Therefore, the author concluded a potential association between periodontitis and breast cancer.’ (page 11)

12.Materials and methods
Page 7 "Potential confounders included level of urbanization". Presumably this is a city / regional level variable in the dataset?
Response: Thanks for your comment. The variable of level of urbanization is an individual level variable based on individual’s residence region in the dataset.

13."Sensitivity analyses were conducted based upon age (<65 years, ≥65 years) and CCI" – further elaboration required.
Response: We are sorry that this is a mistake. “Sensitivity” analyses should be replaced with “Subgroup” analyses. Conducted subgroup analyses and examined the modification effects by age group and CCI group on the association between periodontitis exposure and the risk of female breast cancer. We also added a description regarding the statistical method to assess the modification effect in the statistical method subsection in the Methods section: ‘The significance of modification effect by each covariate on periodontitis exposure-associated female breast cancer risk was examined by calculating the *P*-value of the coefficient associate with the product of each indicator of the covariate and the indicator of periodontitis using the Wald test.’(page 9)

14."We tested the differences in the continuous variables through use of the Student’s t-test and the categorical variables by using the Pearson’s χ2 test" What is the motivation for this and define the specific variables?
Response: We intended to compare the continuous variables and categorical variables between cases and controls. To clarify our motivation of this and define the specific variables, we revised our description as: ‘We compared continuous variables including age and CCI using the Student’s *t*-test and the categorical variables including variables related to periodontitis history and level of urbanization by using the Pearson’s chi-square test between cases and controls.’

15.Logistic regression model – presumably maximum likelihood was used to estimate the model parameters, but it would be good to state this explicitly.
Response: We revised the description regarding statistical analysis in the statistical analysis subsection in the Methods section as:’ A multivariable conditional logistic regression with maximum likelihood estimation was used to examine the association between periodontitis exposure and the risk of breast cancer development after adjustment for potential confounders shown as adjusted odds ratio (aOR) with 95% confidence interval (CI).’

**16.**There is a strong case for modelling the dose response relationship as a continuous response. Arbitrarily creating categories is statistically inefficient and at worst can be misleading. I would advise modelling each explanatory variable of interest for the dose response as a continuous term. This can easily be achieved in SAS using a spline. A good choice would be a restricted cubic spline with the number of knots selected based on prior knowledge or beliefs (3-5 knots is often sensible – see Harrell F. (2015) Regression Modelling Strategies). The dose response adjusted for covariates can be presented as a smooth curve with confidence intervals. Odds ratios can still be obtained from this curve between specific points of interest. You might also consider modelling the CCI this way. Even if this approach is not adopted, the authors might consider mentioning this as a limitation.
Response: We have modelled the dose response relationship of age and CCI for the interaction with periodontitis as a continuous response and showed the results in supplementary figure 1 & 3.

17.Details of the interaction term (later referred to in subsequent sections) in the logistic regression models are missing. This is critical since the primary interest (according to the title of the paper) is effect modification necessitating interaction terms.
Response: Details of the interaction term was described in the Statistical analysis subsection in the Methods section: ‘The significance of modification effect by each covariate on periodontitis exposure-associated female breast cancer risk was examined by calculating the P-value of the coefficient associate with the product of each indicator of the covariate and the indicator of periodontitis using the Wald test.’ (page 9)

18.Perhaps the authors might consider making dataset and SAS code used available as SI
Response: Thank you for your comment. We have provided our dataset and SAS code in the supplementary files.

19.Results
Page 9 "After adjusting for age and index date"…this implies that age and index date were covariates in the regression model – this does not appear to be the case according to the statistical analysis section as it stands.
Response: We are sorry to make a wrong description. Actually, age and year of the index date are matching variables, not adjustment variables. We corrected this description as: ‘After adjusting for CCI and level of urbanization, periodontitis was significantly associated with the risk of female breast cancer (aOR, 1.12; 95% CI, 1.09–1.14).’ (page 9)

‘

20.It would be nice to have some supportive figures (e.g. partial effect plots) summarizing the statistical models.
Response: Thank you for your suggestion. We have conducted partial effect plots to summarize the statistical models and showed them in the supplementary files. (page 10)

21.Discussion
First paragraph talks about several studies one by one – it would be preferable to integrate this as continuous prose and convey the message that your work is supportive of these previous studies.
Response: We had revised our first paragraph of the Discussion section: ‘This is the first nationwide, population-based case control study which assessed the association between a history of periodontitis and the risk of female breast cancer development in Asia, and we have demonstrated a significant association of female breast cancer with periodontitis. The results remained robust in patients aged <65 years or ≥65 years and patients with a CCI = 0. Consistent with our finding, Soder *et al*. [20] selected 3,273 patients aged between 30-40 years and found that chronic periodontitis indicated by missing molars seemed to be associated with breast cancer. Of note, a meta-analysis study [21] found a periodontal disease significantly increased breast cancer risk. However, the risk of breast cancer development was not significantly increased among patients with periodontal disease and a history of periodontal therapy. Therefore, the author concluded a potential association between periodontitis and breast cancer. Freudenheim *et al*. [24] analyzed a cohort of 73,737 postmenopausal women and also revealed that periodontitis was associated with the risk of postmenopausal breast cancer. On the contrary, Farhat *et al*. [25] revealed no association between periodontal disease and overall breast cancer risk (HR 1.02, 95% CI; 0.94–1.10). However, in this study, no confounder was analyzed. In contrast, we analyzed the association between periodontitis and breast cancer adjusting for potential confounds, and we also found the important modification effects by age and comorbidities.’ (page 11)

22."In this study, one important finding is that the risk of breast cancer is modified by both age and comorbidities" - this statement is redundant since it is a repetition of the first sentence of the paragraph.
Response: We deleted this statement. (page 11)

23.Table 3 – It seems patient is the statistical unit as opposed to an adjustment variable as currently indicated at the foot of the table. Also urban should be "urbanization".

Response: We corrected it. (Table 3)
