# Supplementary material for: Stratified analysis of the association between periodontitis and female breast cancer based on age, comorbidities and level of urbanization: A population-based nested case-control study
Source: PLoS One. 2022 Jul 26;17(7):e0271948. doi: 10.1371/journal.pone.0271948 (PMC9321417; doi:10.1371/journal.pone.0271948)
Supplement: Attachment — Submitted filename: response to reviewers.docx [file pone.0271948.s006.docx]

Reviewer #1:

1.The authors have not addressed the comment on how their study was different from the previous study published on periodontal disease and cancer using the same Taiwan database (and also fail to mention this previous study) "A population-based study on the associations between chronic periodontitis and the risk of cancer" Shiu-Dong Chung et al.

Response: Chung et al [20] observed an increased risk of a number of cancers among chronic periodontitis patients, and the adjusted hazard ratio was 1.23 for breast cancer. (page 4)

2.Since breast cancer is a female cancer, stating that they studied females (vs both sexes) makes little sense as obviously prior studies on breast cancer risk were in women only. Please mention this study in the Introduction and this study should not be referred to as the "first" case-control on periodontal disease that is population based (as it is not).
Response: We deleted ‘’first’’. (page 11)

3.This is not a retrospective case-control study. Retrospective case-control studies are studies that recruit cancer patients and ask about their past exposures; the exposure here is measured prior to the development of the disease. So, this is a retrospective cohort study, because the cohort exists and the analysis is conducted on a sample of the cohort retrospectively. Alternatively, you can call it a nested case-control study, which is nested in a cohort population.
Response: We rewrite as ‘’nested case-control study’’. (page 1, 2, 5,11,15)

4.Figure 1 – can you clarify what is meant by “unsured amount” given that half of the population is excluded for this reason (+ missing data) can you justify whether this was a reasonable exclusion and whether it might have caused some selection bias (in other words, how did the excluded population differ from the one included)?
Response: We are sorry to make a mistake here. Actually, we only excluded patients with missing data of residence region (a confounding factor in this study). Also, in 906,287 individuals from the 1997-2013 Longitudinal Health Insurance Database, we excluded excluded 458,873 men first and then excluded anther 26,126 women who had missing data of residence region. We have revised Figure 1.

5.Table 5. The title needs to be modified to clearly describe that the ORs presented are for the comparison of periodontal disease vs no periodontal disease stratified on age, CCI and urbanization. Many readers will misinterpret these results otherwise.
Response: We have revised the tile as ‘Stratified Analysis of the Association Between Periodontitis and Female Breast Cancer Based on Age, Comorbidities and Level of Urbanization: A Population-based Nested Case-control Study.’

6.I don’t think the figures add any value, especially the ones that are based on categorial comparisons.
Response: Thank you for your suggestion. The other reviewer requested us to plot these figures based on both categorical and continuous comparisons. However, we have put them in the supplemental materials.

7.Please edit the abstract for grammar "assess that periodontitis" change to "assess whether periodontitis"

Response: We revised the sentence as: ‘ To conduct stratified analysis of the association between periodontitis exposure and the risk of female breast cancer based on age, comorbidities and level of urbanization.’ (page 2)

Reviewer #2: I enjoyed re-reviewing this interesting study and was pleased that the authors considered many of my suggestions. I only have two minor comments:

8.Response to Reviewers - Reviewer #2: Point 12. The authors responded that "The variable of level of urbanization is an individual level variable based on individual’s residence region in the dataset." I believe this should be termed an ecological level variable since it describes properties of the region, and not each person.
Perhaps a very slight re-wording will suffice.
Response: Thank you for your suggestion. We added a description regarding the variable of level of urbanization ‘The variable level of urbanization is an ecological level variable since it describes properties of the region.’ (page 8)

9.The conclusion section looks a little peculiar with just one sentence. I would add a few more sentences or integrate into the discussion depending on the requirements of the journal.
Response: We revised the conclusion section and integrated it into the Discussion section as: ‘In conclusion, this population-based nested case-control study revealed periodontitis exposure is significantly associated with the risk of breast cancer. We also found this association is modified by both age and comorbidities. Further studies are warranted to clarify the mechanisms.’(page 15)
